# Lenvatinib Combined With a PD-1 Inhibitor as Effective Therapy for Advanced Intrahepatic Cholangiocarcinoma

**DOI:** 10.3389/fphar.2022.894407

**Published:** 2022-06-01

**Authors:** Lulu Xie, Jingzheng Huang, Linling Wang, Wenrui Ren, Hao Tian, Anhong Hu, Jun Liang, Yuqing Jiao, Yali Li, Qunfang Zhou, Wenjing Zhang

**Affiliations:** ^1^ Department of Minimally Invasive Interventional Radiology and Department of Radiology, the Second Affiliated Hospital of Guangzhou Medical University, Guangzhou, China; ^2^ Department of Ultrasound, the Second Affiliated Hospital of Guangzhou Medical University, Guangzhou, China

**Keywords:** advanced intrahepatic cholangiocarcinoma, lenvatinib, PD-1 inhibitor, combination therapy, chemotherapy failure

## Abstract

**Background:** Lenvatinib combined with a PD-1 inhibitor has obtained a satisfactory antitumor effect in several solid tumors. However, the efficacy and tumor response of lenvatinib with a PD-1 inhibitor in advanced intrahepatic cholangiocarcinoma still need further exploration.

**Methods:** This is a single-arm study for the assessment of the efficacy and tolerability of lenvatinib with a PD-1 inhibitor in intrahepatic cholangiocarcinoma patients who had chemotherapy failure. Efficacy was evaluated based on the Response Evaluation Criteria in Solid Tumors RECIST Version 1.1 (RECIST 1.1).

**Results:** A total of 40 patients with advanced intrahepatic cholangiocarcinoma were enrolled after the chemorefractory effect. The median progression-free survival was 5.83 ± 0.76 months. The 3-month and 6-month progression-free survival rates were 80.0% and 32.5%, respectively. The median overall survival was 14.30 ± 1.30 months. The 12-month and 18-month overall survival rates were 61.4% and 34.7%. The 3-month RECIST 1.1 evaluation was that seven patients (17.5%) showed partial response, 23 patients (57.5%) had stable disease, and 10 patients (25.0%) had progressive disease. The objective response rate was 17.5%, and the disease control rate was 75.0%. All the recorded any-grade adverse events inducing treatment termination were controllable, and there were no AE-related deaths.

**Conclusion:** Our study showed that a combination of lenvatinib with the PD-1 inhibitor could be an effective treatment for advanced intrahepatic cholangiocarcinoma after the chemorefractory effect.

## Introduction

Intrahepatic cholangiocarcinoma (ICC) is a hepatobiliary tumor with a high death rate, which presents an unsatisfied prognosis with 10% of 5-year overall survival (OS) and a median OS of approximately 24 months (Kelley et al., 2020). ICC ranks second and accounts for approximately 10% of primary liver malignancy (Chun and Javle, 2017). The symptoms of ICC are insidious and nonspecific which include abdominal discomfort, weight loss, indigestion, or asymptomatic elevation of liver functions on routine laboratory testing, and only a minority of patients are diagnosed at an early stage with the tumor removed by surgery (Esnaola et al., 2016). Therefore, most patients present in the advanced stage will require effective systemic therapy. Currently, the established first-line treatment was gemcitabine and cisplatin, and the second-line treatment was FOLFOX systemic chemotherapy (Rizvi et al., 2018). However, the efficacy of these approaches is still unsatisfactory, and patients easily develop the chemorefractory effect (Moeini et al., 2016). There is now no uniform therapy for advanced ICC after chemotherapy failure. The shortage of available therapeutic regimens has plagued the oncologists exploring new strategies.

Lenvatinib is an oral tyrosine kinase inhibitor that restrains the vascular endothelial growth factor receptor (VEGFR) 1–3, fibroblast growth factor receptors (FGFR) 1–4, and platelet-derived growth factor receptor (Kudo et al., 2018). Due to the advantage of inhibiting tumors with multiple pathways, this multitargeted tyrosine kinase inhibitor is being used for the treatment of many tumors (Hao and Wang, 2020). Immunotherapy has emerged as a major tool in cancer treatment with the recent success of trials with PD-1/PD-L1 axis blockade (Balar and Weber, 2017). Programmed death-1 (PD-1) is a checkpoint molecule on T cells, which plays a vital role in controlling tumor progression through immune responses (Balar and Weber, 2017). Studies have proved that a combination of therapies involving lenvatinib and the PD-1 inhibitor could produce a synergetic effect, and lenvatinib with the PD-1 inhibitor has an augment antitumor effect than alone (Kimura et al., 2018). This combination of lenvatinib and the PD-1 inhibitor now has been applied for the treatment of many cancers including hepatocellular carcinoma, renal cell carcinoma, thyroid cancer, and endometrial carcinoma (Motzer et al., 2015; Schlumberger et al., 2015; Finn et al., 2020; Makker et al., 2020). The combination of lenvatinib and a PD-1 inhibitor is efficacious and promising, and the combination is considered to be a good pair of active drugs in malignancy therapy (Wang et al., 2019).

Given these factors, this combination could be an effective treatment for advanced ICC and prolong the survival of patients. Lin J et al. reported that lenvatinib with pembrolizumab was promising in alternative patients with refractory bile tract carcinoma, and the therapeutic outcomes were delightful as a non–first-line treatment (Lin et al., 2020). Ding Y et al. reported that chemotherapy, tislelizumab, and lenvatinib could be an effective therapeutic regimen for preoperative advanced intrahepatic ICC conversion therapy (Ding et al., 2021). These studies have inspired clinical investigations of applying the regimen in patients with advanced ICC. However, studies that reported lenvatinib with a PD-1 inhibitor on advanced ICC are few, so there is still a necessity for clinical evidence to further obtain knowledge of this combination therapy. In this report, we focused on lenvatinib combined with a PD-1 inhibitor in patients with advanced ICC after chemotherapy failure.

## Materials and Methods

This study was conducted in accordance with the principles of the Declaration of Helsinki (World Medical Association, 2013), and the study protocol was approved by the Ethics Committee of the Second Affiliated Hospital of Guangzhou Medical University (no.2022-hg-ks-12).

### Study Population

We retrospectively reviewed the medical records of patients who received a diagnosis of advanced ICC from June 2018 to June 2020 at the Second Affiliated Hospital of Guangzhou Medical University. Patients who met the following criteria were included in this study: 1) histologically confirmed ICC; 2) all patients experienced disease progression or could not tolerate systematic therapy; 3) at least one measurable tumor lesion according to the RECIST 1.1 criteria; 4) Eastern Cooperative Oncology Group (ECOG) performance status of 0–1; 5) patients who had adequate liver function (i.e., Child–Pugh class A or B liver function); 6) had adequate renal coagulation function; and 7) age 18–75 years. The exclusion criteria were as follows: 1) patient intolerance to lenvatinib or the PD-1 inhibitor; 2)death or missed the follow-up within 3 months; 3) inadequate liver or kidney function; and 4) patients who received other tyrosine kinase inhibitor with or without PD-1 inhibitor.

The tumor stage was assessed by systemic imaging (either enhanced computed tomography (CT) of the chest or bone scan, contrast-enhanced CT or magnetic resonance imaging (MRI) of the abdomen or brain, or positron emission tomography/computed tomography (PET/CT). Baseline levels of liver function and blood tests were collected. The albumin–bilirubin (ALBI) grade for each patient was calculated using the formula: ALBI score = (log10 bilirubin × 0.66) + (albumin × −0.085). The ALBI grade is used to identify different mortality risk subsets of patients as follows: grade 1 (lowest mortality risk) for ALBI score ≤ −2.60, grade 2 (intermediate mortality risk) for ALBI score > −2.60 and ≤ −1.39), and grade 3 (highest mortality risk) for ALBI score > −1.39 (Hiraoka et al., 2019).

### Treatment and Assessment of the Response

All patients accepted contrast material–enhanced CT or MRI within 2 weeks before lenvatinib administration. Information regarding the information of initiation, completion of treatment, initial dose, dose modifications, and adverse events (AEs) during treatment was systematically collected. The prescription dosage of lenvatinib was 12 mg (for patients with a bodyweight ≥60 kg) or 8 mg (for patients with a bodyweight <60 kg) orally once a day. For the PD-1 inhibitor, the PD-1 inhibitor (tislelizumab) dose was applied according to the drug instructions.

### Follow-Up

The follow-up period for this study was terminated on 30 June 2021. Laboratory tests including CA-125, albumin, bilirubin, aspartate transaminase (AST), alanine transaminase (ALT), and prothrombin time (PT) were performed to evaluate the treatment response and liver function every six weeks after treatment. Patients were evaluated at least once every six weeks after treatment. Each follow-up visit involved performing screening abdominal imaging (e.g., abdominal, chest, bone, brain CT, and/or MRI). Target tumors were selected to a maximum of two lesions per organ and five lesions in total. The minimum size for measurability is greater than 1 cm. The tumor imaging response was evaluated according to the Response Evaluation Criteria in Solid Tumors version 1.1 (Schwartz et al., 2016). In brief, the complete response (CR) was defined as the disappearance of arterial enhancement in the tumor. Partial response (PR) was defined as ≥30% shrinking in the diameter of the targeted tumors. Progressive disease (PD) was defined as at least a 20% increase in the sum of the diameter of the targeted tumors or the appearance of a new lesion. Stable disease (SD) neither met the CR nor PR and PD. The primary endpoint for the study was overall survival (OS), and the secondary endpoint was progression-free survival (PFS). OS was defined as the time from accepting lenvatinib and the PD-1 inhibitor to death or the last follow-up, and the PFS was defined as the time from the date of accepting lenvatinib and the PD-1 inhibitor to tumor progression or the last follow-up.

### Statistical Analysis

The data were presented as a summary of the baseline characteristics, therapeutic efficacies, and AEs. The 3- and 6-month PFS and 6-, 12-, and 18-month OS were all estimated by the Kaplan–Meier method. The hazard ratio (HR) of each clinical factor was estimated by Cox proportional hazard modeling.

## Results

### Patient Characteristics

**FIGURE 1:**
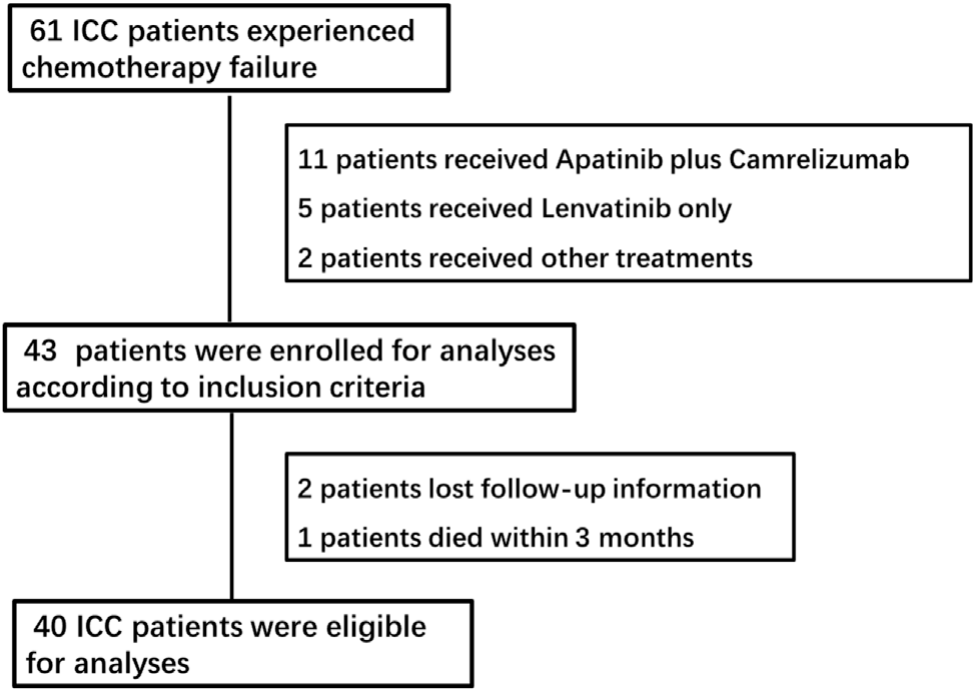
Flow chart of the study. Efficacy and safety of the treatment.

A total of 61 patients were enrolled for drug administration, and 40 patients were included for analyses (Figure 1). The median patient age was 53.0 years (range, 43.0–58.8), and 31 patients (77.5%) were males, and nine patients (22.5%) were females. In total, 28 (70.0%) patients had an ECOG performance status of 1, and 16 patients (40.0%) had HBV infection. All patients with hepatitis received regular antiviral therapy during lenvatinib and PD-1 inhibitor treatment. A total of 30 (72.5%) patients had poor tumor differentiation, and 32 (80.0%) patients had metastases, including intrahepatic, lymph nodes, lung, and bone metastases. A total of 25 patients (62.5%) received local therapy or surgery before lenvatinib and PD-1 inhibitor treatment (Table 1).

**TABLE 1:** Baseline characteristics of patients in the entire cohort.

Parameter	Total
Age, years (median, IQR)	53.0 (43.0–58.8)
Gender, n [%]	9 [22.5]
Female	31 [77.5]
Male	
Differentiation	11 [27.5]
Moderate	29 [72.5]
Poor	
ECOG status	12 [30.0]
0	28 [70.0]
1	
ALBI grade	31 [77.5]
1	9 [22.5]
2	
Hepatitis	16 [40.0]
Metastasis, n [%]	28 [70.0]
Intrahepatic	26 [65.0]
Lymph nodes	17 [42.5]
Lungs	8 [20.0]
Bone	
Previous therapy	13 [32.5]
Surgery	40 [100.0]
Systemic chemotherapy	8 [20.0]
Thermal ablation	14 [35.0]
Transarterial chemoembolization	
Macrovascular tumor thrombus	10 [25.0]
Tumor size, cm, (median, IQR)	6.7 [4.9–8.2]
CA-199, U/ml, (median, IQR)	13.2 [13.2–299.7]

**FIGURE 2:**
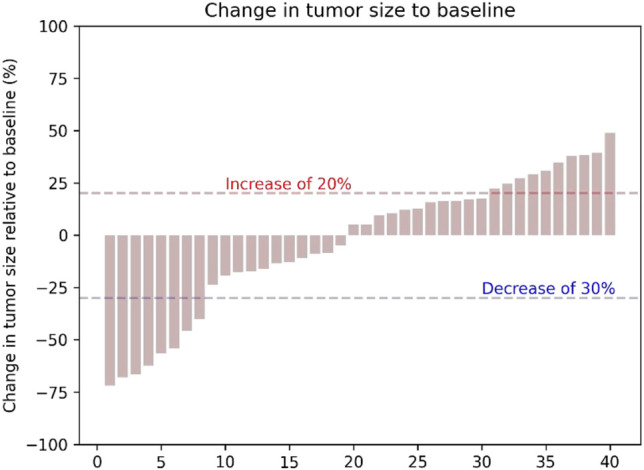
Tumor percentage changes from the baseline in terms of the target lesion sizes.

**FIGURE 3:**
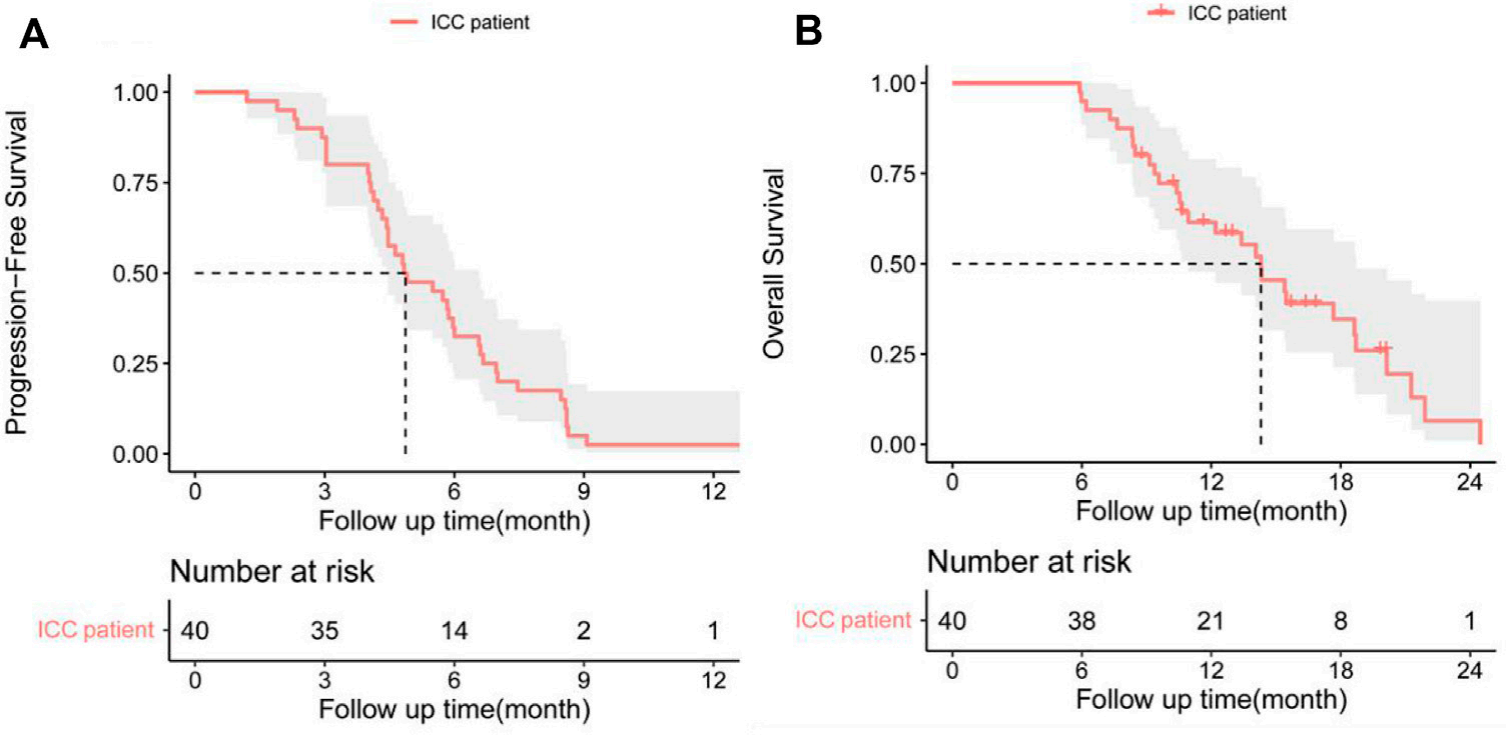
**(A)** Progression-free survival (PFS) and **(B)** overall survival (OS) in patients with advanced intrahepatic cholangiocarcinoma after the chemorefractory effect.

In the cohort, all patients had a regular follow-up, and the clinical responses were assessed. Overall, 19 of the 40 (47.5%) patients exhibited a decrease in the tumor size from the baseline (Figure 2). The median progression-free survival (PFS) was 5.83 ± 0.76 (95% CI, 4.34–7.33) months (Table 2). The 3-month and 6-month PFS rates were 80% and 32.5% (Figure 3A), respectively. In total, 11 patients were still alive during the follow-up period. The median overall survival (OS) was 14.30 ± 1.30 (95%: 11.76–16.84) months (Table 2). The 12-month and 18-month OS rates were 61.4% and 34.7% (Figure 3B). The 3-month RECIST 1.1 evaluation was that seven (17.5%) patients showed partial response (PR), 23 (57.5%) had stable disease (SD), and 10 (25.0%) had progressive disease (PD). The objective response rate (ORR) was 17.5%, and the disease control rate (DCR) was 75.0% (Table 2). We further determined the clinical benefit rate (CBR, PFS≥ 6 months) in all assessment-available patients. The CBR was 32.5% (Table 2).

**TABLE 2:** Therapeutic efficacy of the response and survival outcome of patients treated with lenvatinib with the PD-1 inhibitor.

Therapeutic response assessment	Evaluation of patients (*n* = 40)
Objective response rate (ORR, %)	7 (17.5%)
Disease control rate (DCR, %)	30 (75.0%)
Complete response (CR, %)	0
Partial response (PR, %)	7 (17.5%)
Stable disease (SD, %)	23 (57.5%)
Progressive disease (PD, %)	10 (25.0%)
Clinical benefit rate (%)	15 (32.5%)
Progression-free survival (median, 95% CI, months)	4.83 ± 0.68 (3.49–6.18)
Overall survival (median, 95% CI, months)	14.30 ± 1.30 (11.76–16.84)

All the recorded any-grade adverse events (AEs) inducing treatment termination were controllable, and there were no AE-related deaths. The most common AEs (any grade) were decreased appetite, hypertension, fatigue, and diarrhea (Table 3). For AE grade ≥3, the most common were hypertension and proteinuria (Table 3). Most AEs occurring during treatment were safe and tolerated.

**TABLE 3:** Most common treatment-related adverse events in patients receiving lenvatinib and the PD-1 inhibitor.

Adverse events	Grades 1 and 2	Grades 3 and 4
Decreased appetite	19 (47.5)	0
Hypertension	18 (45.0)	3 (7.5)
Fatigue	13 (32.5)	1 (2.5)
Diarrhea	11 (27.5)	0
Increased ALT/AST	9 (22.5)	1 (2.5)
Proteinuria	9 (22.5)	2 (5.0)
Hypothyroidism	7 (17.5)	0
Rash or desquamation	7 (17.5)	0
Weight decreased	5 (12.5)	0

### PFS and OS Analysis

Cox-regression analysis results regarding the prognostic factors of PFS or OS were further analyzed. Univariate analysis revealed that intrahepatic metastases, lymph nodes metastases, lung metastases, bone metastases, and ALBI grade 2 were correlated with poorer PFS. Multivariate analysis illustrated that intrahepatic metastasis (HR = 3.08, 95% CI: 1.23–7.67, and *p* = 0.016) and ALBI grade 2 (HR = 3.84, 95% CI: 1.42–10.03, and *p* = 0.005) were related to poorer PFS (Table 4). Univariate analysis revealed that intrahepatic metastases, lymph nodes metastases, lung metastases, and bone metastases were correlated with poorer OS. Multivariate analysis illustrated that intrahepatic metastasis (HR = 9.02, 95% CI: 1.80–45.07, and *p* = 0.007) was related to poorer OS (Table 5).

**TABLE 4:** Univariate and multivariate analyses of prognostic factors on progression-free survival (PFS) in 40 patients with advanced ICC after chemotherapy failure.

Variable	Univariate and multivariate analyses				
	Comparison	HR (95% CI)	*P*	HR (95% CI)	*P*
Tumor differentiation	Moderate vs. poor	1.09 (0.53–2.25)	0.816		
Macrovascular invasion	No vs. yes	2.34 (0.42–1.82)	0.723		
Intrahepatic metastases	**No vs. yes**	**2.34 (1.15–4.7)**	**0.019**	**3.08 (1.23–7.67)**	**0.016**
Lymph nodes metastases	**No vs. yes**	**2.72 (1.35–5.48)**	**0.005**	1.95 (0.86–4.40)	0.109
Lung metastases	**No vs. yes**	**5.03 (2.13–11.84)**	**< 0.001**	1.97 (0.76–5.15)	0.165
Bone metastases	**No vs. yes**	**2.49 (1.06–5.85)**	**0.037**	1.47 (0.61–3.50)	0.390
ALBI grade	**1 vs. 2**	**3.94 (1.71–9.07)**	**0.001**	**3.84 (1.42–10.03)**	**0.005**
ECOG status	0 vs. 1	0.74 (0.37–1.47)	0.387		
Hepatitis	No vs. yes	1.42 (0.74–2.74)	0.289		
Surgery	Yes vs. no	1.35 (0.68–2.65)	0.388		
TACE	Yes vs. no	0.92 (0.48–1.79)	0.811		
Ablation	Yes vs. no	0.92 (0.42–2.02)	0.842		
Sex	Female vs. male	1.36 (0.64–2.89)	0.432		
Smoking	No vs. yes	1.22 (0.63–2.36)	0.564		

Bold values means the P<0.05.

**TABLE 5:** Univariate and multivariate analyses of prognostic factors on overall survival (OS) in 40 patients with advanced ICC after chemotherapy failure.

Variable	Univariate and multivariate analyses				
	Comparison	HR (95% CI)	*P*	HR (95% CI)	*P*
Tumor differentiation	Moderate vs. poor	1.42 (0.62–3.25)	0.404		
Macrovascular invasion	No vs. yes	0.92 (0.40–2.11)	0.847		
Intrahepatic metastases	**No vs. yes**	**5.32 (2.01–14.15)**	**0.001**	**9.02 (1.80–45.07)**	**0.007**
Lymph node metastases	**No vs. yes**	**2.66 (1.11–6.38)**	**0.029**	1.67 (0.67–4.29)	0.269
Lung metastases	**No vs. yes**	**3.31 (1.40–7.83)**	**0.006**	1.90 (0.75–5.13)	0.173
ALBI grade	1 vs. 2	0.49 (0.22–1.11)	0.088		
ECOG status	0 vs. 1	0.94 (0.40–2.18)	0.876		
Bone metastases	No vs. yes	1.32 (0.49–3.58)	0.579		
Hepatitis	No vs. yes	1.98 (0.88–4.49)	0.101		
Surgery	Yes vs. no	1.46 (0.62–3.40)	0.386		
TACE	Yes vs. no	1.50 (0.70–3.22)	0.294		
Ablation	Yes vs. no	0.67 (0.26–1.72)	0.407		
Sex	Female vs. male	1.55 (0.72–3.31)	0.261		
Smoking	No vs. yes	0.92 (0.49–1.72)	0.784		

Bold values means the P<0.05.

## Discussion

The therapeutic strategy for advanced ICC is challenging worldwide as the ICC usually indicates a poor prognosis. Surgical resection is the only potentially curative treatment for ICC; however, the 5-year OS rate was 15–40% (Weber et al., 2015). For advanced or recurrent ICC, the first-line treatment was chemotherapy. However, patients usually developed refractory; then, the second-line therapy was varied and disappointed (Sirica et al., 2019). PD-1 inhibitor-based immune therapy has obtained significant improvement in several tumors, including melanoma, lung cancer, and head and neck malignancies (Sui et al., 2018; Gavrielatou et al., 2020; Guo et al., 2020). However, immune monotherapy faces many challenges in biliary cancer. Studies showed that the efficacy of the PD-1 inhibitor alone in biliary cancer remains unsatisfactory (Ueno et al., 2019). In the Makoto et al. study, combined therapy of biliary tract cancer (nivolumab PD-1 inhibitor and chemotherapy) achieved obvious better benefits than the PD-1 inhibitor alone (Ueno et al., 2019).

Combining the strategy with antiangiogenic molecular target drugs or chemotherapy could improve the efficacy of immunotherapies and has shown promising clinical results (Rizvi et al., 2018; Wang et al., 2019). Mei K et al. have reported that camrelizumab combined with apatinib has achieved promising results in the treatment of advanced ICC. The medium PFS and OS were 1.9 and 13.4 months (Mei et al., 2021). These results were superior to the previously reported efficacy of apatinib alone in ICC (Hu et al., 2020). Lin J et al. reported that lenvatinib with pembrolizumab was considered a non-first-line therapy in treating refractory bile tract carcinoma, and this study obtained ORR which was 25%, and the DCR was 78.1%. The median PFS was 4.9 months, and the 6-month PFS rate was 33.7%. The median OS was 11.0 months, and the 1-year OS rate was 39.4% (Lin et al., 2020). In our study, the ORR was 17.5%, and the DCR was 75.0%. The median PFS and 6-month PFS rates were 5.8 months and 32.5%. The median OS was 14.3 months, and the 12-month and 18-month rates were 61.4%. Our results were in accordance with the research of Lin et al. (2020).

Ueno M et al. demonstrated lenvatinib as monotherapy for advanced biliary tract cancer, and the ORR was 11.5%. The median PFS was 3.19 months, and the median OS was 7.35 months (Ueno et al., 2020). Our study was better than Ueno M’s results. Lenvatinib combined with PD-1 inhibitors have provided new ideas for advanced ICC. Previous studies have proved that lenvatinib could enhance the antitumor efficacy of PD-1 inhibitors by restraining angiogenesis (Shigeta et al., 2020). Thus, the combination of these two agents is promising and satisfactory when they are used in patients with refractory ICC. Compared with the previous reports, our study focused on ICC with chemotherapy failure, and patients were in a more advanced stage than the published literature. We found that the combination therapy of lenvatinib and the PD-1 inhibitor was effective and competent for ICC patients in a more advanced stage. A well-designed prospective trial with other second-line treatments is needed to determine the precise efficacy and safety of this combination therapy, or a further trial of this combined regimen with chemotherapy as first-line therapy is promising.

However, there were some limitations to our study; first, it was a retrospective study, so there is a need to develop a prospective trial to evaluate this combination therapy as second-line therapy in advanced ICC. Second, the small sample of our study limits more information on factors related to the prognosis; further reports with more patients and a multicenter study are needed to get more comprehensive results. Third, this study was the real-world application of lenvatinib and the PD-1 inhibitor, and it is impossible to exclude interference from the doctor and patients in terms of treatment.

In summary, our research provides evidence that a combination of lenvatinib with the PD-1 inhibitor could be an effective treatment for ICC after the chemorefractory effect. This combination could achieve controllable safety and good efficacy, thereby providing a new treatment option for advanced ICC.

## Data Availability

The raw data supporting the conclusion of this article will be made available by the authors, without undue reservation.
